# Effects of intra-fourth ventricle injection of crocin on capsaicin-induced orofacial pain in rats

**Published:** 2015

**Authors:** Esmaeal Tamaddonfard, Sina Tamaddonfard, Salar Pourbaba

**Affiliations:** 1 *Division of Physiology, Department of Basic Sciences, Faculty of Veterinary Medicine, Urmia University, Urmia, Iran*; 2 *Faculty of Veterinary Medicine, Urmia Azad University, Urmia, Iran*

**Keywords:** *Crocin*, *Morphine*, *Naloxone*, *Orofacial pain*, *Rats*

## Abstract

**Objectives:**

Crocin, a constituent of saffron and yellow gardenia, possesses anti-nociceptive effects. In the present study, we investigated the effects of intra-fourth ventricle injection of crocin in a rat model of orofacial pain. The contribution of opioid system was assessed using intra-fourth ventricle injection of naloxone, an opioid receptor antagonist.

**Materials and Methods:**

A guide cannula was implanted into the fourth ventricle of brain in anesthetized rats. Orofacial pain was induced by subcutaneous (s.c.) injection of capsaicin (1.5 µg/20 µl) into the right vibrissa pad. The time spent face rubbing/grooming was recorded for a period of 20 min. Locomotor activity was measured using an open-field test.

**Results:**

Intra-fourth ventricle injection of crocin (10 and 40 µg/rat) and morphine (10 and 40 µg/rat) and their co-administration (2.5 and 10 µg/rat of each) suppressed capsaicin-induced orofacial pain. The analgesic effect induced by 10 µg/rat of morphine, but not crocin (10 µg/rat), was prevented by 20 µg/rat of naloxone pretreatment. The above-mentioned chemical compounds did not affect locomotor activity.

**Conclusion:**

The results of this study showed that the injection of crocin into the cerebral fourth ventricle attenuates capsaicin-induced orofacial pain in rats. The anti-nociceptive effect of crocin was not attributed to the central opioid receptors.

## Introduction

Crocin is the major yellow pigment in gardenia yellow and saffron (Lee et al., 2005 ▶; Aung et al., 2007 ▶). This compound has several pharmacological effects such as antioxidant, anti-cancer, anti-diabetic, anti-epileptic and neuroprotective properties (Aung et al., 2007 ▶; Tamaddonfard et al., 2012 ▶; Tamaddonfard et al., 2013a ▶; Alavizadeh and Hosseinzadeh, 2014 ▶; Asri-Rezaei et al., 2015 ▶). Recent experimental studies have suggested important roles for crocin in modulation of pain. For example, intraperitoneal (i.p.) injection of crocin suppressed the second phase of formalin-induced orofacial pain (Erfanparast et al., 2015 ▶). Moreover, intracerebroventricular (i.c.v.) injection of crocin reduced thenumber of eye wipes in acute corneal pain induced by hypertonic saline in rats (Tamaddonfard and Hamzeh-Gooshchi, 2010b ▶).

Orofacial pain refers to pain associated with the soft and hard tissues of the head, face and neck. It is a common problem in the population that has profound sociologic effects and a considerable impact on quality of life (De Ross, 2013 ▶). Trigeminal nerve relays noxious stimuli information to the higher regions and nuclei of the brain such as brain stem trigeminal complex, thalamus, hippocampus and cerebral cortex (Takemura et al., 2006 ▶; Takeda et al., 2011 ▶). It has been shown that the opioid system may be involved in the local peripheral, spinal and supraspinal modulation of orofacial pain (Eisenberg et al., 1996 ▶; Duale et al., 1996 ▶; Tamaddonfard et al., 2011 ▶; Tamaddonfard et al., 2014 ▶; Erfanparast et al., 2015 ▶). The orofacial capsaicin test in rats was introduced by Pelissier et al. (2002) ▶, and has been frequently used in the study of orofacial pain and analgesic mechanisms (Pelissier et al., 2002 ▶; Holanda Pinto et al., 2008 ▶; Quintans-Junior et al., 2010 ▶).

This study was designed to investigate the effects of crocin injection into the fourth ventricle of the brain on capsaicin-induced orofacial pain in rats. Because of the importance of opioid system in the orofacial pain modulation (Tamaddonfard et al., 2011 ▶; Tamaddonfard and Hamzeh-Gooshchi, 2010b ▶), the involvement of central opioid receptors in the anti-nociceptive effect of crocin was evaluated using an opioid receptor antagonist, naloxone. 

## Materials and Methods

**Animals**

Healthy adult male Wistar rats (250-280 g) were used in this study. The animals were maintained in the Rat House of Laboratory of Physiology in Faculty of Veterinary Medicine, Urmia University, Urmia, Iran under controlled 12h-12h light-dark cycle and ambient temperature (22 ± 0.5°C) and food and water were available* ad libitum*. All experiments were performed between 12:00 and 17:00. The research and animal care procedures were approved by the Veterinary Ethics Committee of Faculty of Veterinary Medicine of Urmia University and were performed in accordance with the National Research Council Guide for Care and Use of Laboratory animals (NRC, 2011).

**Chemicals**

The following chemicals were used: crocin (Fluka, Germany), morphine sulfate (Temad, Iran), capsaicin and naloxone hydrochloride (Sigma-Aldrich, USA). Capsaicin was dissolved in ethanol/dimethyl sulfoxide (DMSO)/distilled water (1:1:8 v/v/v) (Holanda Pinto et al., 2008 ▶). Other chemicals were dissolved in normal saline 30 min before intra-fourth ventricle administration.

**Animal grouping**

In a pilot study, normal saline, vehicle (ethanol/DMSO/distilled water) and capsaicin were injected subcutaneously into the vibrissa pad to compare the severity of pain-related behavior among them. Thereafter, 78 rats were divided into 13 groups with six rats in each group. Group 1 received normal saline (i.c.v.); Groups 2, 3 and 4 received crocin at the doses of 2.5, 10, and 40 µg/rat, i.c.v., respectively; Groups 5, 6 and 7 received morphine at the doses of 2.5, 10, and 40 µg/rat, i.c.v., respectively; Group 8 received crocin (2.5 µg/rat, i.c.v.) + morphine (2.5 µg/rat, i.c.v.); Group 9 received crocin (10 µg/rat, i.c.v.) + morphine (10 µg/rat, i.c.v.); Groups 10 and 11 received naloxone at the doses of 10 and 20 µg/rat, i.c.v., respectively; Groups 12 and 13 received naloxone (20 µg/rat, i.c.v.) + crocin (10 µg/rat, i.c.v.) and naloxone (20 µg/rat, i.c.v.) + morphine (10 µg/rat, i.c.v.), respectively. Doses that were used here were in accordance with previous investigations (Kahveci et al., 2006 ▶; Hamurtekin et al., 2007 ▶; Tamaddonfard and Hamzeh-Gooshchi, 2010b ▶). 

**Surgery**

To deliver the chemical agents into the brain, a permanent guide cannula was implanted in the fourth ventricle of the brain. In brief, each rat was anaesthetized with i.p. injection of a mixture of ketamine (80 mg/kg) and xylazine (10 mg/kg), and a 23-gauge, 12-mm stainless-steel guide cannula was stereotaxically (Stoelting Stereotaxic Apparatus, Wood Dale, IL, USA) placed in the fourth ventricle of the brain. The stereotaxic coordinates, according to Paxinos and Watson (1997) ▶, were: -12.5 mm posterior to the bregma, 0 mm lateral to the midline and 7.8 mm below the top of the skull. The guide cannula was anchored with two screws and dental acrylic. A 12-mm stylet was inserted into the guide cannula to keep it patent prior to injection. All animals were allowed to recover from surgery for 10 days.

**Intra-fourth ventricle injection**

Intra-fourth ventricle injections of drugs and controls were performed using a 13-mm length injection needle connected via a 20-cm polyethylene tube to a 5-μl Hamilton syringe. Intracerebral injection was performed over a period of 30 s with a total volume of 1 µl. After completion of each injection, the injection needle was left in place for a further 30 s to facilitate diffusion of the drug solution. Naloxone was injected 8 min before induction of orofacial pain, whereas this period for crocin and morphine was 5 min. In the case of co-administration, crocin and morphine were injected 8 and 5 min before pain induction, respectively. We used intra-fourth ventricle injection procedure because descending inhibitory and facilitatory pain pathways of orofacial pain such as rosteroventromedial nucleus (RVMN) are located in the structures like medulla oblongata and pons near the fourth ventricle (Vanegas and Schaible, 2004 ▶; Ossipov et al., 2010 ▶; Bourne et al., 2014 ▶). 

**Orofacial capsaicin test**

For induction of orofacial pain, each rat was placed in a plexiglass observation chamber (30 cm × 30 cm × 30 cm), and after a 30-min adaptation period, capsaicin (1.5 µg/20 µl) was subcutaneously injected into the left vibrissa using a 27-gauge injection needle. The time spent of face rubbing/grooming wasrecorded over a period of 20 min. The volume and dose of capsaicin used here were in accordance to Pessilier et al. (2002) ▶. The observers were blinded to the used chemicals. 

**Locomotor activity**

Locomotor activity was assessed in an open-field test as described previously (Hamzeh-Gooshchi et al., 2015; Erfanparast et al., 2015). The apparatus consisted of a wooden box (120 cm × 120 cm × 50 cm). The floor of the arena was divided into 16 equal squares. To monitor the activity, animals were placed directly in one corner of the open field apparatus. The number of squares crossed with all paws (horizontal movement) and the number of rearing (vertical movement) were counted in a 5-min session. 

**Verification of cannula**

At the end of each experiment, 1 µl of methylene blue was injected into the fourth ventricle. Animals were euthanized with high-dose ether, and the brains were removed and placed in a formalin solution (10%). After 24 h, the brains were sectioned coronally (50-100 µm) and viewed under a loup to observe the distribution of methylene blue in the fourth ventricle according to the atlas of Paxinos and Watson (1997) ▶. 

**Statistical analysis**

Statistical comparisons were performed using GraphPad Prism (Version 5) software (GraphPad Software, San Diego, CA, USA). One-way ANOVA and then Tukey’s test were applied to compare the differences among experimental groups. In figures, all values are expressed as mean ± SEM. A value of p<0.05 was considered statistically significant.

## Results

**Figure 1:**
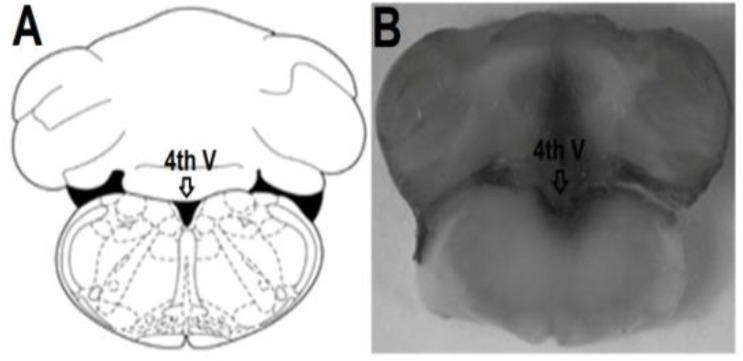
Schematic illustration of transverse section of the rat brain that shows the location of the fourth ventricle (A).Location of the permanent cannula site in the fourth ventricle (arrow) of all rats included in the data analysis (B). Atlas plate adopted from Paxinos and Watson (1997), 4th V: fourth ventricle

The placements of the tip of the cannula in the fourth ventricle of rats are shown in Figure 1.

The rat brain section was adopted from the atlas of Paxinos and Watson, (1997) ▶ (Figure 1A). The location of the cannula tip placement in the fourth ventricle was shown with arrow (Figure 1B).

**Figure 2:**
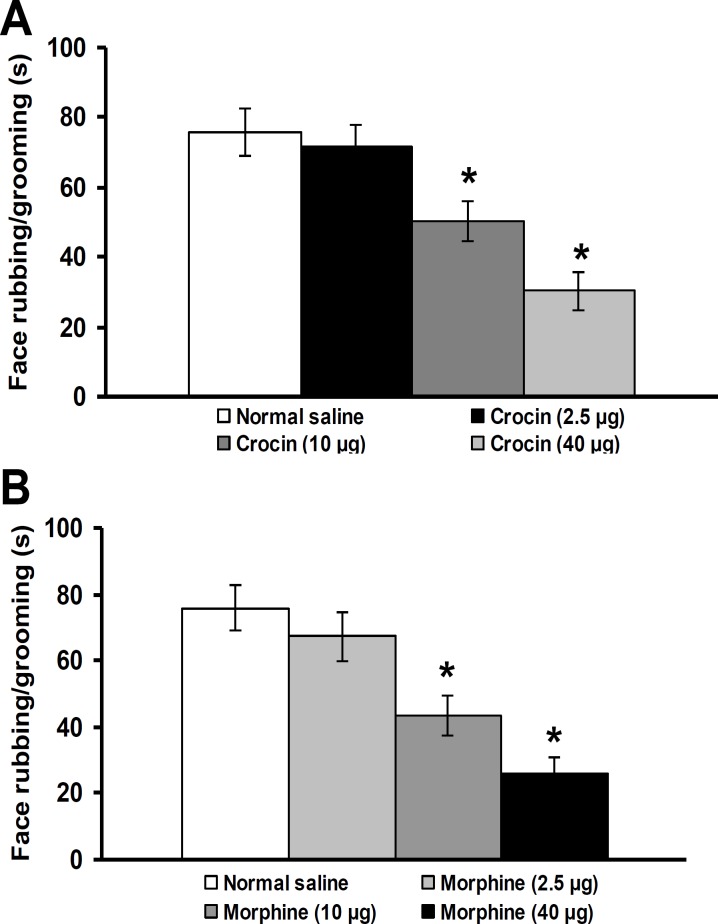
Effects of intra-fourth ventricle injection of crocin (A) and morphine (B) on face rubbing/grooming duration induced by subcutaneous injection of capsaicin in vibrissa pad. All values are expressed as mean ± SEM (n=6). * indicates a significant difference at p<0.05 in comparison with normal saline treated group

**Figure 3:**
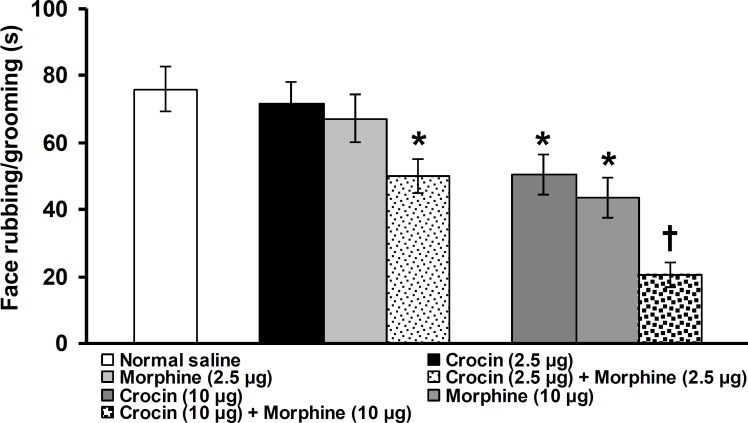
Effects of intra-fourth ventricle combined injection of low and medium doses of crocin and morphine on face rubbing/grooming duration induced by subcutaneous injection of capsaicin in vibrissa pad. All values are expressed as mean ± SEM (n = 6). * indicates a significant difference at p<0.05 in comparison with normal saline treated group, † indicates a significant difference at p<0.05 in comparison with crocin (10 µg/rat), morphine (10 µg/rat) and crocin (2.5 µg/rat) + morphine (2.5 µg/rat) treated groups

Intra-fourth ventricle injection of crocin (2.5 µg/rat) and morphine (2.5 µg/rat) did not change the severity of pain, whereas crocin (10 and 40 µg/rat) ( p<0.05, Figure 3A) and morphine (10 and 40 µg/rat) (p<0.05, Figure 3B) significantly reduced face rubbing/grooming durations. No significant differences were observed between the anti-nocicepptive effects of crocin and morphine (Figure 3).

**Figure 4:**
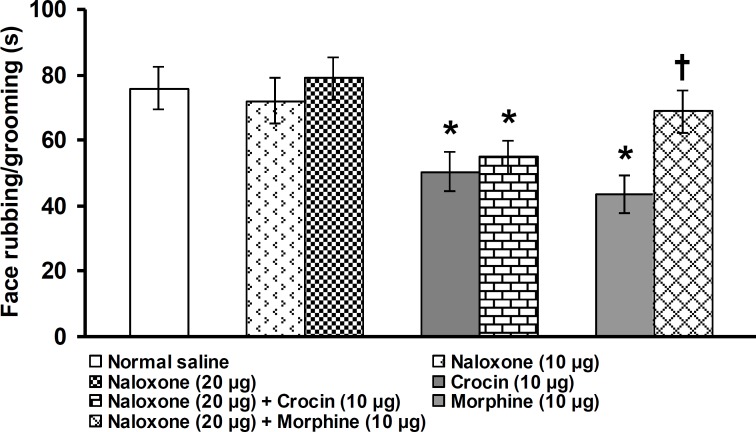
Effects of intra-fourth ventricle administration of naloxone alone and its administration before crocinand morphine on face rubbing/grooming duration induced by subcutaneous injection of capsaicin in vibrissa pad. All values are expressed as mean ± SEM (n = 6). * indicates a significant difference at p<0.05 in comparison with normal saline treated group, † indicates a significant difference at p<0.05 in comparison with morphine (10 µg/rat) treated group

In co-administration mode, intra-fourth ventricle injection of crocin (2.5 µg/rat) and morphine (2.5 µg/rat) significantly decreased pain-related behavior when compared with normal saline injected group (p<0.05, Figure 4). By increasing the dose to 10 µg/rat of each chemical compound, a significant suppressive effect was observed in comparison with 10 µg/rat of each chemical compound used alone (p<0.05, Figure 4).

Intra-fourth ventricle of naloxone alone at the doses of 10 and 20 µg/rat did not change the severity of pain. Pretreatment with naloxone (20 µg/rat) did not inhibit crocin (10 µg/rat)-induced anti-nociception, whereas significantly prevented the anti-nociceptive effect of morphine (10 µg/rat) (p<0.05, Figure 5).

Intra-fourth ventricle injection of the above-mentioned chemical compounds did not influence the number of lines crossing and rearing in the open-field test (data not shown). 

## Discussion

The present study showed that s.c. injection of capsaicin into the vibrissa pad resulted in face rubbing/grooming behavior in rats. Capsaicin, the pungent ingredient of hot peppers which is applied to skin, muscle and joints has been shown to be able to produce inflammation, activate and sensitize spinal and trigeminal small-diameter afferents, evoke nociceptiove behavior in animals, and cause intense pain, hyperalgesia and referred pain in humans (Tang et al., 2004 ▶; Arendt-Nielsen et al., 2008 ▶; Honda et al., 2014 ▶). The s.c. injection of capsaicin (1.5 µg/20 µl) into the vibrissa pad produced face rubbing/grooming behavior in rats (Pelissier et al., 2002 ▶; Holanda et al., 2008 ▶). Our results confirm the findings of previous studies (Pelissier et al., 2002 ▶; Holanda et al., 2008 ▶). 

Also, our results showed central anti-nociceptive effects for crocin, morphine and their co-administration. There are no reports showing the central effects of crocin on capsaicin-induced inflammatory pain. In other tests for inflammatory pain including hind paw and orofacial formalin tests, it was found that systemic administration of crocin produced anti-nociceptive effects (Tamaddonfard and Hamzeh-Gooshchi, 2010a ▶; Erfanparast et al., 2015 ▶). In a rat model of carrageenan-induced hyperalgesia (Tamaddonfard et al., 2013b ▶), and also in spinal cord injury-induced chronic pain (Karami et al., 2013 ▶), systemic administration of crocin suppressed pain-related behaviors. Only in one study, it has been reported that lateral ventricle injection of crocin at doses of 12.5, 25 and 50 µg reduced the number of eye wipes in a rat model of acute trigeminal pain (Tamaddonfard and Hamzeh-Gooshchi, 2010b ▶). Morphine administered either systemically (in the neck) or locally (in the vibrissa pad) dose-dependently reduced face rubbing/grooming behavior provoked by s.c. injection of capsaicin in rats (Pellisier et al., 2002 ▶). Similar anti-nociceptive result was obtained after s.c. injection of morphine (Holanda Pinto et al., 2008 ▶). In addition, i.c.v. injection of morphine produced a potent analgesia in patients with intractable pain from trigeminal neuralgia or cluster headache (Lee et al., 2014 ▶). Therefore, morphine can modulate local peripheral, spinal and supraspinal mechanisms of pain. The results of present study showed that co-administration of crocin (2.5 µg/rat) and morphine (2.5 µg/rat) produced an anti-nociceptive effect in rats (group 8). The anti-nociceptive effect of co-administration of crocin and morphine was potentiated by increasing the dose, so that the orofacial pain was further attenuated by co-administration of higher doses of crocin and morphine (10 µg/rat, i.c.v., group 9). This finding is in agreement with previous studies in which systemic co-injection of crocin and morphine produced anti-nociceptive effect in formalin-induced hind paw and orofacial pain in rats (Tamaddonfard and Hamzeh-Gooshchi, 2010a ▶; Erfanparast et al., 2015 ▶). In the acute corneal pain evoked by local corneal application of hypertonic saline, i.p. and i.c.v. injection of crocin increased the anti-nociceptive effect of i.p. morphine (Tamaddonfard and Hamzeh-Gooshchi, 2010b ▶). 

The results of the present study showed that pretreatment with naloxone prevented morphine-induced, but not crocin-induced, antinociception. This means that opioid receptor is not involved in the antinociceptive effect of crocin. Naloxone is a competitive antagonist of µ, and κ opioid receptors with higher affinity for µ opioid receptor (Helm et al., 2008 ▶). It has been frequently used for the assessment of opioid system involvement in mediating pain and analgesia (Helm et al., 2008 ▶; Trescot et al., 2008 ▶). In the hind paw and orofacial formalin test in rats, s.c. injection of naloxone did not prevent crocin-induced antinociception, whereas it reversed the anti-nociceptive effect of morphine (Tamaddonfard and Hamzeh-Gooshchi, 2010a ▶; Erfanparast et al., 2015 ▶). In addition, using an acute model of trigeminal pain in rats, the anti-nociceptive effect induced by i.p. and i.c.v. injection of crocin was not reversed by i.p. injection of naloxone (Tamaddonfard and Hamzeh-Gooshchi, 2010b ▶). These findings indicate that peripheral as well as central opioid receptor is not involved in crocin-induced antinociception.

The antinociception induced by separate and combined intra-fourth ventricle injection of crocin, morphine and naloxone observed in the present study, may be associated with the effects of these compounds on nociceptive pathways, because the locomotor activity was not affected. It is well known that commonly used analgesics including opioids, steroidal and non-steroidal anti-inflammatory drugs, anti-epileptics and serotonin-norepinephrine reuptake inhibitors produce adverse effects which interrupt their analgesic effects (Carter et al., 2014 ▶). However, i.p. injection of crocin at a high dose of 400 mg/kg reduced cage crossing, sniffing, rearing and grooming behaviors (Tamaddonfard and Hamzeh-Gooshchi, 2010a ▶). 

In conclusion, the results of the present study showed that injection of crocin into the cerebral fourth ventricle attenuates capsaicin-induced orofacial pain in rats. The anti-nociceptive effect of crocin was not attributed to central opioid receptor**s.**
